# Advancing molecular macrobenthos biodiversity monitoring: a comparison between Oxford Nanopore and Illumina based metabarcoding and metagenomics

**DOI:** 10.7717/peerj.19158

**Published:** 2025-04-14

**Authors:** Karlijn Doorenspleet, Amalia Aikaterini Mailli, Berry B. van der Hoorn, Kevin K. Beentjes, Annelies De Backer, Sofie Derycke, Albertinka J. Murk, Henning Reiss, Reindert Nijland

**Affiliations:** 1 Marine Animal Ecology, Wageningen University and Research, Wageningen, Netherlands; 2 Faculty of Biosciences and Aquaculture, Nord University, Bodø, Norway; 3 Naturalis Biodiversity Center, Leiden, Netherlands; 4 ILVO Marine Research, Flanders Research Institute for Agriculture, Fisheries and Food, Oostende, Belgium

**Keywords:** North Sea, Benthic communities, DNA, Metabarcoding, Metagenomics, Nanopore, NovaSeq, Illumina, Miseq

## Abstract

DNA-based methods and developments of sequencing technologies are integral to macrobenthos biodiversity studies, and their implementation as standardized monitoring methods is approaching. Evaluating the efficacy and reliability of these technological developments is crucial for macrobenthos biodiversity assessments. In this study, we compared three DNA-based techniques for assessing the diversity of bulk macrobenthos samples from the Belgian North Sea. Specifically, we compared amplicon sequencing using Illumina MiSeq and portable real-time sequencing of Oxford Nanopore *versus* shotgun sequencing using Illumina NovaSeq sequencing. The 313 bp mitochondrial cytochrome c oxidase subunit I (COI) metabarcoding fragment served as the target region for the metabarcoding analysis. Our results indicate that Oxford Nanopore and MiSeq metabarcoding had similar performances in terms of alpha and beta diversity, revealing highly similar location-specific community compositions. The NovaSeq metagenomics method also resulted in similar alpha diversity, but slightly different community compositions compared to the metabarcoding approach. Despite these differences, location-specific community compositions were maintained across all platforms. Notably, read counts from the NovaSeq metagenomic analysis showed the weakest correlation to size corrected morphological abundance and there were mismatches between morphological identification and all DNA based findings which are likely caused by a combination of factors such as primer efficiency and an incomplete reference database. Our findings underscore the critical importance of database completeness prior to implementing DNA-based techniques as standardized monitoring method, especially for metagenomics. Nevertheless, our findings emphasize that Oxford Nanopore metabarcoding proves to be a viable alternative to the conventional Illumina MiSeq metabarcoding platform for macrobenthos biodiversity monitoring.

## Introduction

The European Union established the Marine Strategy Framework Directive (MSFD, 2008/56/EC), alongside the European Water Framework Directive (WFD, 2000/60/EC) and the European network for protected areas for conserving the most valuable species and habitats (Natura 2000 network, Habitat Directive). These directives form the basis of ecosystem management and use a variety of monitoring-based assessments (*e.g.*, Borja et al., 2010) that aim to inform (non-) governmental decisions on marine environmental health. However, taxonomy-based monitoring practices rely on great taxonomic expertise, are time-consuming and limited in taxonomic resolution (Danovaro et al., 2016; Pawlowski et al., 2018), particularly when identifying understudied taxonomic groups and species at different life stages (von Ammon et al., 2018; Gleason, Hanner & Cottenie, 2023). Consequently, DNA-based technological developments have gained particular interest for their potential in studying marine biodiversity (Bucklin, Steinke & Blanco-Bercial, 2011; Cordier et al., 2019; Elbrecht et al., 2017; Lanzén et al., 2017; Leray & Knowlton, 2016). A reduction in labour time and the growing reference sequence libraries demonstrate DNA based methods to be a promising alternative for monitoring (Baird & Hajibabaei, 2012; DeSalle & Goldstein, 2019; Gostel & Kress, 2022; Leray & Knowlton, 2015). Different DNA-based monitoring studies have demonstrated correspondence with morphological findings (Derycke et al., 2021; Mauffrey et al., 2021). As a result, DNA-based methods are repeatedly proposed as a suitable routine biodiversity assessment strategy to inform policy (Aylagas et al., 2020; Goodwin et al., 2017; Hering et al., 2018; Hinz et al., 2022; Pawlowski et al., 2018; Thalinger et al., 2019).

Generally, DNA-based biodiversity assessments rely on DNA metabarcoding, which enables the identification of species from environmental samples by amplifying a short DNA fragment using universal primer pairs. This amplified DNA is then sequenced with next-generation sequencing platforms to identify the taxa found in samples (Taberlet et al., 2012). As biases can be introduced at each stage (Van der Loos & Nijland, 2021), decisions need to be made on the sampling method and the appropriate use of preservation techniques (Gaither et al. , 2011; Ransome et al., 2017), DNA extraction methods, using sufficient replicates (Van den Bulcke et al., 2021), and using appropriate primer pair(s) (Creer et al., 2016; Devloo-Delva et al., 2018; Leray & Knowlton, 2016). Bioinformatics pipelines are also crucial to take into consideration as the pipeline choice, and the processing settings greatly influence the output (Antich et al., 2021).

Current developments in DNA-based methods have resulted in the availability of several different third-generation sequencing platforms, such as Illumina, Ion Torrent, Oxford Nanopore sequencing and Pacific Biosciences (Hu et al., 2021). These platforms provide exciting opportunities to study the environment in convenient ways that include obtaining abundance data (Klunder et al., 2022), epigenetic modifications (Zhao, Van Bodegom & Trimbos, 2023) and population genetics (Jahnke et al., 2022). For metabarcoding of gene fragments shorter than 500 bp, Illumina technologies are currently the standard platform because of its high accuracy (Meyer & Kircher, 2010). In comparison to Illumina MiSeq, the Oxford Nanopore sequencing platform measures an electrical current that is produced when the nucleotides of a sequence pass through a transmembrane nanopore, allowing for real-time sequencing and base calling (Bleidorn, 2016; Wang et al., 2021). The advantages of Oxford Nanopore sequencing include lower costs, the sequencing of long fragments and its suitability for real-time in-field experiments (Krehenwinkel, Pomerantz & Prost, 2019). However, the error rates of raw Oxford Nanopore sequences are currently higher (87–99% accuracy) compared to Illumina platforms, therefore different bioinformatics processing pipelines have been used to circumvent this problem (Baloğlu et al., 2021; Doorenspleet et al., 2025; Egeter et al., 2020). Short read Oxford Nanopore sequencing has previously been shown to be consistent with Illumina MiSeq in low-diversity samples (Egeter et al., 2020; Van Der Reis et al., 2023). However, comparisons of the sequencing platforms with high diversity samples were only performed using bacterial communities, and show a lack of several taxa (Heikema et al., 2020; Stevens et al., 2023). Thus, it remains unclear to what extent short read Oxford Nanopore sequencing is directly comparable to Illumina data.

Recently, metagenomics sequencing have gained interest as alternatives to metabarcoding for community analysis (Bernatchez et al., 2024; Theissinger et al., 2023). Shotgun sequencing can bypass some methodological disadvantages that are inherent to metabarcoding such as PCR (Zhou et al., 2013) and primer amplification bias (Leray & Knowlton, 2015), because DNA is directly processed for sequencing. Moreover, shotgun sequencing has been suggested to cover the full spectrum of biota in a sample and provide a correlation with morphological biodiversity studies (Monchamp et al., 2022). This method is seen as a viable contestant to metabarcoding methods to monitoring genetic diversity (Bista et al., 2018; Lopez et al., 2022; Monchamp et al., 2022). However, metagenomics can be limited by (i) the sequencing depth, which has become more cost-efficient with the advent of current sequencing platforms such as NovaSeq, and (ii) the availability and quality state of reference sequences within widely used databases (Weigand et al., 2019), given that the shotgun sequencing process is non-selective. Thus, it is inconclusive whether shotgun sequencing is currently more useful for both diversity detection and relative abundance data for macrobenthos studies.

In this study, we compared different DNA-based methods: paired end Illumina MiSeq metabarcoding, Oxford Nanopore MinION metabarcoding of the 313 bp cytochrome c oxidase subunit I (COI) mitochondrial fragment and Illumina NovaSeq Metagenomics shotgun sequencing. We used bulk macrobenthos community samples collected from different soft-bottom habitats along the Belgium North Sea. By using different DNA-based methods and sequencing platforms for metabarcoding, we assessed the suitability of these methods for monitoring benthic community composition and diversity. For the metabarcoding methods, we used a curated COI North Sea invertebrate reference database and compared it to a metagenomics method using the complete NCBI-nt database. We hypothesized that the metabarcoding data from both sequencing platforms and the shotgun metagenomics method are generally robust and resemble the morphologically identified community both in alpha and beta diversity.

## Materials and Methods

### Sample collection

**Figure 1:**
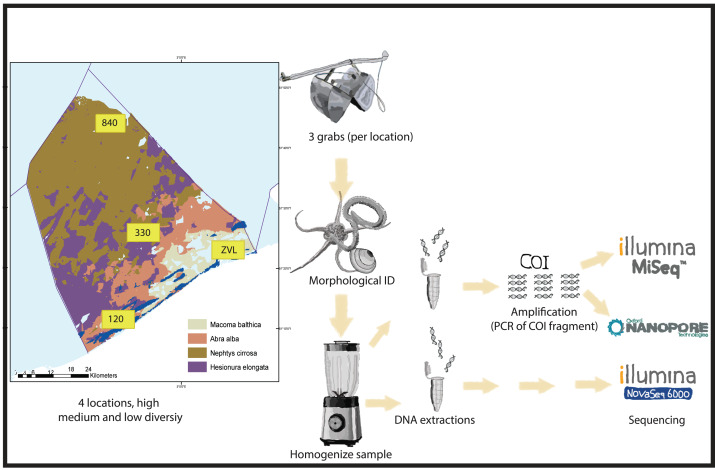
Experimental set-up. Graphical presentation of methods used in this study. Samples were taken in the Belgian part of the North Sea with known macrobenthos communities (see Breine et al., 2018 for details). The samples were taken in triplicates using a van Veen grab on 4 different locations with high medium and low biodiversity. The animals found in the samples were either morphologically identified or homogenized for DNA extraction. For both amplicon based and metagenomics, different subsamples were used for DNA extraction. Depending on DNA based method, either a COI 313 bp fragment was amplified (Nanopore or Illumina MiSeq) or DNA was directly sequenced with Illumina Novaseq shotgun sequencing for metagenomics.

Sampling was conducted at four locations in the Belgian North Sea that contained different macrobenthos communities with high, medium, and low diversity (see Breine et al., 2018) (Fig. 1). The bulk samples have been previously used to optimize the metabarcoding protocols and to test the method reproducibility (Derycke et al., 2021; Van den Bulcke et al., 2021; Van den Bulcke et al., 2023). Samples were collected from a coastal muddy fine-sand habitat with a high taxa diversity of sessile tube-forming organisms and high bioturbation (location 120—*Abra alba* community). Samples were also collected from a medium sand habitat with a medium taxa diversity of mobile organisms (location 330—*Nepthys cirrosa* community), a coarse sand habitat with high taxa diversity for sessile interstitial species (location 840—*Hesionura elongata* community) and lastly, a muddy habitat with low taxa diversity (location ZVL—*Macoma balthica* community) (Breine et al., 2018). A Van Veen grab (with a sampling surface area of 0.1 m^2^) was used to collect three biological replicates per location (A, B, C). On average, each sample consisted of 9.3 Liters of material. All sediment samples were sieved using a one mm sieve, and the remaining material (for example, shells and rocks) was fixed using absolute ethanol and stored at −20 °C prior to further processing.

### Morphological identification

The morphological identification followed the protocols described by Derycke et al. (2021) and Van den Bulcke et al. (2021); Van den Bulcke et al. (2023). Organisms from one replicate per location (120-B, 840-C, 330-C, ZVL-A) were identified to species level and juveniles to genus level, except for specimens belonging to Nemertea, Anthozoa and Oligochaeta, which were identified up to phylum, subphylum, and order level, respectively. The complete list of species identified in each location is available (Table S1). Species were recorded per individual hence no biomass information was available for this dataset. To correct for the lack of biomass data, the count data were multiplied by the average size (from each size class). This was obtained to correlate morphology abundance data and the read count of each DNA based method for the identified species.

### DNA extraction

For molecular comparison, all specimens isolated from each field replicate were retained to obtain a bulk sample. Bulk samples were homogenized with a blender or, if the sample was <100 ml, with a mortar and pestle. Subsamples of six ml were taken and stored in Eppendorf tubes at −20 °C before processing in different institutes (Table S2). Samples used for Illumina MiSeq and Oxford Nanopore sequencing were extracted at Naturalis Biodiversity Centre (Leiden, The Netherlands) and used by Wageningen University and research (Wageningen, The Netherlands) for amplification and sequencing with Oxford Nanopore. Samples used for Illumina NovaSeq were processed at Nord University (Bodø, Norway). For every location, three subsamples of two ml were used for DNA extraction. For location 840C, ZVLA and ZVLC, DNA extract from ILVO was used (Table S2, Oostende, Belgium) due to low recovery of sample material. All institutes (Nord, Naturalis and ILVO) used the same extraction protocol according to Van den Bulcke et al. (2023). In short, the Eppendorf tubes were centrifuged for 3 min at 10,000 RPM, and the supernatant was removed. Samples were incubated at 50 °C for 1 h to evaporate the remaining ethanol. Three subsamples from each biological replicate (3X3) were incubated overnight at 56 °C in the power-beads tube of the DNeasy PowerSoil Kit (Qiagen, Hilden, Germany), supplemented with 10 µL of proteinase K (20 mg/ml).

The DNA extracted from each subsample was pooled and cleaned using the Wizard DNA clean-up system (Promega, Madison, WI, USA) and eluted in 50 µL TE buffer. After processing, samples were stored at −20 °C before amplification or shotgun sequencing (Fig. 1).

### PCR amplification

For amplification, a 313 bp COI fragment was amplified using the forward mlCOIlintF primer (Leray et al., 2013) with the reverse jgHCO2198 primer (Geller et al., 2013) in combination with Oxford Nanopore and Illumina MiSeq extension sequence (printed red in Table 1). However, a modified version of the primers was used by replacing deoxy-inosines (I) with degenerated bases (N) (Table 1). Amplification was performed on each sample in triplicate. Each reaction contained 8.5 µL nuclease-free water, 12.5 µL 2x KAPA HiFi HotStart ReadyMix (Roche, Basel, Switzerland), 0.75 µL (10 µM) forward and 0.75 µL (10 µM) reverse primer and 2.5 µL of DNA template. For Oxford Nanopore sequencing, DNA template was diluted 10x prior to amplification. PCR conditions were 3 min at 95 °C, 35 cycles of 30 s at 98 °C, 30 s at 57 °C, 30 s at 72 °C and a final extension for 1 min at 72 °C. PCR replicates were pooled, and a clean-up was performed using a 2:1 mixture with AMPURE beads (Beckman Coulter Inc., Brea, CA, USA) and >70% ethanol. Amplification was confirmed using gel electrophoresis (1% gel, ethidium bromide).

**Table 1:** Primers used for metabarcoding. Primers used for the Illumina MiSeq and Nanopore metabarcoding method. Sequences indicated in bold are the platform specific adapters to the primers.

Oxford nanopore		
Forward	miCOIintF: 5′- ** TTTCTGTTGGTGCTGATATTGC**GGWACWGGWTGAA CWGTWTAYCCYCC-3′	Leray et al. (2013)
reverse	jgHCO2198:5′-**ACTTGCCTGTCGCTCTATCTTC**TANACYTCNGGRTGNCCRA ARAAYCA-3′	Geller et al. (2013)
Illumina Miseq		
Forward	miCOIintF: 5′-**TCGTCGGCAGCGTCAGATGTGTATAAGAGACAG**GGWACWGG WTGAACWGTWTAYCCYCC-3′	Leray et al. (2013)
reverse	jgHCO2198:5′-**GTCTCGTGGGCTCGGAGATGTGTATAAGAGACAG**TANA CYTCNGGRTGNCCRAARAAYCA-3′	Geller et al. (2013)

### Illumina MiSeq metabarcoding

#### Index PCR

For the index PCR, five µL nuclease-free water, 12.5 µL 2X KAPA HiFi HotStart ReadyMix (Roche, Basel, Switzerland) and 2.5 µL of each index primer (Nextera XT primer 1 and 2) without modifications was used with 2.5 µL of initial pooled PCR product. The PCR program was 3 min at 95 °C, 8 cycles of 30 s at 95 °C, 30 s at 55 °C, 30 s at 72 °C and a final extension for 3 min at 72 °C. Amplification was confirmed using gel-electrophoresis (1% gel, ethidium bromide). The purified Index PCR products were equimolarly pooled and sequenced using the Illumina MiSeq 2X300bp platform (v2), with an addition of 20% of PhiX (sequenced by Baseclear BV).

#### Bioinformatics of Illumina MiSeq reads

After Illumina MiSeq sequencing, the quality of the demultiplexed reads was checked using *MultiQC* (Ewels et al., 2016), and primers were removed using *Trimmomatic* (Bolger, Lohse & Usadel, 2014). Amplicon sequence variants (ASV) were generated using the *DADA2* pipeline in the *Dada2* v1.17.0 package (Callahan et al., 2016) in R Studio v4.0.2 (R Core Team, 2021). Standard settings were used and an error rate of three mismatches was allowed. Reads with a quality score lower than 30 were removed. Unique paired-end reads were determined, merged, and filtered for chimeras for each sample with the removeBimeraDenovo function using the *Dada2* v1.17.0 package (Callahan et al., 2016). Taxonomy was assigned using the *assignTaxonomy* function in the *Dada2* package (Wang et al., 2007) using the *Ribosomal Database Project* (RDP) (Wang et al., 2007) with a minimum bootstrap confidence parameter of 80.

### Reference database for metabarcoding methods

A morphologically curated database that contains 1,992 COI sequences of 565 North Sea invertebrate species was used for taxonomic identification (http://dx.doi.org/10.5883/DS-GEANS1). This database was assembled from public and an in-house marine macrobenthos COI sequences that were obtained during multiple diversity monitoring campaigns throughout the North Sea (https://northsearegion.eu/geans/about/). During these campaigns, missing species were actively collected, vouchered and barcodes when possible.

### Oxford Nanopore metabarcoding

#### Oxford Nanopore sequencing

The PCR barcoding kit 96 (EXP-PCB096) was used for the barcoding PCR, and the sequence library was prepared with the SQK-LSK114 kit (Oxford Nanopore Technologies, UK). Several adaptations deviated from the manufacturer’s instructions: barcoding PCR was achieved in a total volume of 10 µL using 0.3 µL 10 µM PCR barcode primer pair and 10–50 ng amplicon. The following PCR program was used: initial denaturation at 95 °C for 3 min, 15 cycles of 95 °C for 10 s, 62 °C for 15 s, 65 °C for 90 s, followed by a final extension at 65 °C for 180 s.

The concentration of the barcoded PCR products was measured using the Qubit HS kit (Thermo Fisher Scientific, Waltham, MA, USA) on the non-purified products, after which barcoded PCR products were pooled in equimolar ratios. The pooled amplicon sequence library was cleaned twice using AMPURE beads (Beckman Coulter Inc., Brea, CA, USA). The first clean-up step used 70% ethanol and the second used Short Fragment Buffer (SFB) to enrich for the target size fragments. After end prep and adapter ligation, the library was again washed with SFB during the final clean-up of the protocol. A total of five µL library containing 98.5ng DNA was loaded onto an R10.4.1 flow cell (Oxford Nanopore Technologies, UK) mounted on a Minion Mk1C device.

#### Oxford Nanopore sequence read processing

Oxford Nanopore sequence read processing was performed according to the post-processing protocol as described by Doorenspleet et al. (2025). Basecalling of the fast5 pass files was performed using *Guppy* (Version 6.5.7, Oxford Nanopore Technologies, UK) in super high accuracy (SUP) mode. The *Decona* pipeline was used (https://github.com/Saskia-Oosterbroek/decona) for trimming, clustering, and taxonomic assignment of the reads. Raw base-called reads were trimmed to between 250–400 bases for each sequence. A cluster similarity of 85% was set as the clustering threshold of the sequences. Medaka consensus sequences were generated from each cluster larger than five reads (Decona -f -q 10 -T 18 -l 300 -m 320 -c 0.85 -g “GGWACWGGWTGAACWGTWTAYCCYCC;max_error_rate=0.1;min_overlap=20... TANACYTCNGGRTGNCCRAARAAYCA;max_error_rate=0.1;min_overlap=19” -n 5 -r 0.99 -R 500 -k 6 -M).

### Taxonomic assignment

The consensus sequences were classified using *BLASTn* (NCBI, version 2.11.0), prior to further processing, the classifications were manually checked on inconsistent classifications. For taxonomic identification, the Top five hits were considered for each consensus sequence. Of these, the best match was used for taxonomic assignment based on hits with the highest e-value. The best match was assigned to species level when there was a minimal alignment length of 250 nucleotides with <4 bp mismatches and >98% identity. The best match was assigned to genus level with >97% identity, family level with >95% identity, >93% for order level and >90% for phylum level identification. The North Sea invertebrate species reference database was used for taxonomic identification (http://dx.doi.org/10.5883/DS-GEANS1). This database was the same as used for the Illumina MiSeq metabarcoding method, but not for the metagenomics method.

### Read abundance correction

After taxonomic assignments, a tag correction was performed on the Oxford Nanopore data to correct the tag jumping that had occurred. Tag jumping had occurred when both forward and reverse barcode tags were on sequences that did not belong to that barcode. After troubleshooting, most of the contamination could be alleviated by removing 1% of the total read count of each species from each barcode. This correction is intended to correct proportionally for the total read abundance of each taxon. Although no positive control sample was used for this study, PCR negative controls did not show any positive band nor higher DNA concentrations after barcode PCR. However, the raw data contained a low amount of reads of the most commonly occurring species in these. As the same species also occurred in the ZVL samples with low read abundance where we did not expect them based on the Illumina results, we decided to remove these reads by a minimum 1% read abundance correction. Therefore, we decided to remove these reads by a 1% read abundance correction and this appeared to reduce these observations.

First, the total read count was calculated for all species in all barcodes using the mutate() function in the *dplyr* v1.1.0 package. Second, 1% of the total read count was calculated and rounded to a whole number of reads. Finally, 1% of the total read count of each species was subtracted from each barcode.

### NovaSeq shotgun sequencing

#### Preparations for sequencing

After DNA extraction and clean-up, libraries were directly prepared using NEBNext^®^ Ultra™ II DNA Library Prep Kit for Illumina (New England Biolabs, Ipswich, MA, USA). Samples were indexed using NEBNext^®^ Multiplex Oligos for Illumina (New England Biolabs, Ipswich, MA, USA). The final quality check was conducted using the Agilent Tapestation system using hds1000 screen tape (Agilent Technologies, Santa Clara, CA, USA). Samples were pooled in equimolar concentrations and sent to the Norwegian Sequencing Center in Oslo to be sequenced using the NovaSeq S4 quarter flow cell (2x150).

### Sequence read processing

The NovaSeq reads were trimmed using cutadapt (–minimum-length = 100, *q* = 30), and merged with *PEAR v 1.7.2* (Zhang et al., 2014) using the default parameters. Reads were then assembled using idba_ud (Peng et al., 2012) using default parameters. Reads were mapped back to the contigs using BBMap (http://sourceforge.net/projects/bbmap/) with the parameter -minid set to 90. BLASTn was used to align the contigs with settings set at >97% identity, *e*-value set at −10, and alignment length equal and over 100 bp. Taxonomic assignments to more than one phylum per query were removed and then the match with the highest bit-score was kept for the data analysis. Single alignments were discarded for the data analysis.

### Reference databases for metagenomics method

To compare the performance of different databases, contigs were locally aligned to four sets of databases. One, the curated COI database for North Sea specific marine invertebrates (GEANS v4, http://dx.doi.org/10.5883/DS-GEANS1). Two, the metagenomics data were also aligned a manually curated NCBI-nt database (original complete NCBI-nt downloaded on 22.05.2022) that mainly contained marine eukaryotes. Three, the nuclear entries of the curated NCBI-nt database only, and four, the mitochondrial entries of the curated NCBI-nt database only. The complete NCBI-nt database including both nuclear and mitochondrial entries gave the most optimal result, and therefore this database was used for the comparison with the metabarcoding methods.

### Data analysis

Data analysis was carried out in RStudio v4.2.2 (R Studio v4.2.2). A rarefaction curve (*Vegan* v2.6.4) was plotted to understand the effect of differential sequencing depths between samples. Each sample showed a flattening curve (Fig. S1), which indicated that for each DNA-based method, an appropriate sequencing depth was achieved. Therefore, the data were not rarefied but transformed using a log_10_ transformation. All reads from the metabarcoding method (Illumina MiSeq and Oxford Nanopore) were compared on ASV /consensus sequence level to verify the initial assignment between sequences (>97% percentage identity). Phylum level comparisons were made to determine the differences between general taxonomic composition between the different DNA based methods (Miseq, Nanopore, NovaSeq). For visualisation of the database comparison with the Novaseq results, a Pie chart was used (ggplot2, v3.4.0). Species level richness, and Shannon diversity index (H, log 10 transformed) of the read counts were calculated using the diversity() function (*Vegan* v2.6.4) and visualized using boxplot (ggplot2, v3.4.0). Normality of the data was tested using Shapiro–Wilk for normal distribution and Q-Q plots. Based on these results a 2-way ANOVA using the aov() function (stats, v3.6.2) was carried out to determine the differences between sampling locations and the DNA-based method used and whether an interaction effect could be observed. For a pairwise comparison, a *post/hoc* analysis was performed using the Tuckey test (HSD) using the TukeyHSD() function (stats, v3.6.2). For beta diversity, non-metric multidimensional scaling (‘bray’) was performed on each dataset separately (Oxford Nanopore metabarcoding, Illumina MiSeq metabarcoding and Shotgun metagenomics sequencing) in combination with *betadisper* to check for homogeneity of variance. A PERMANOVA was used along with a pairwise *post-hoc* analysis using a Bonferroni correction (both available in adonis2(), *Vegan* v2.6.4) to analyse which locations were significantly different from each other, within each dataset. A Spearman rank correlation was used (stats v3.6.2), to compare the size class corrected morphological abundance findings with each DNA-based method.

## Results

### Read processing comparison between DNA-based methods

From the Illumina MiSeq metabarcoding samples, a total of 10,206,087 sequences were obtained with 8,692,409 used after processing. Of these, 8,330,994 reads were used as ASVs for taxonomy assignment (39 species). An overview is available of the sequencing output of each DNA-based method after sequencing and processing (Table S3). From the Oxford Nanopore metabarcoding dataset, 2,426,017 reads were basecalled. This is a lower sequencing depth than commonly used for Oxford Nanopore sequencing (Van Der Reis et al., 2023). However as indicated, the lower sequencing depth was not reflected in the rarefaction curves indicating that it was not informative to sequence any further. Of the basecalled reads, 1,841,385 remained after clustering and consensus building. A total of 1,191,853 of the remaining reads were used as consensus sequences for taxonomy (48 species). From the 3,060,417,120 shotgun reads obtained from the Illumina NovaSeq metagenomics run, 2,425,520,473 reads passed the quality values for direct taxonomic assignment of the reads. For the species assignment using the COI curated invertebrate database, 42,262 reads (39 species) could be assigned to species level whereas the complete NCBI database with marine eukaryotes was used, a total of 8,512,483 (36 species) reads could be assigned to species level, which represented 0.35% of the total filtered reads. For direct comparison of each species for each dataset see further.

### Initial comparison and Novaseq metagenomics reference database choice

Direct comparison to the processed metabarcoding sequences between Oxford Nanopore (consensus sequences) and Illumina MiSeq (ASVs), indicated that most of the sequences were similar between methods (Fig. S2). In addition, phylum level assignment showed that both the number of phyla as well as the proportion of phyla is very similar between metabarcoding methods (Fig. S3).

**Figure 2:**
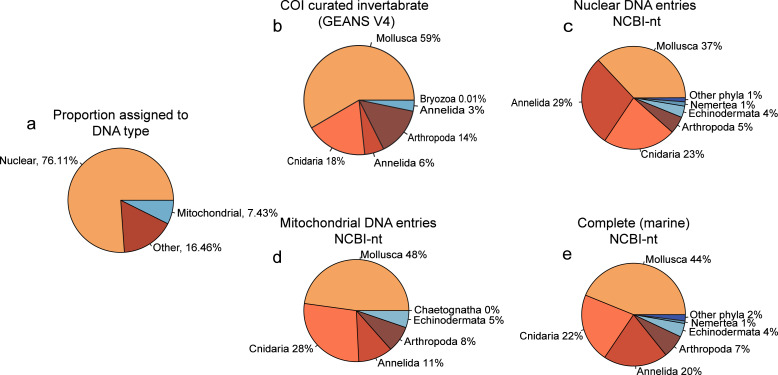
Proportion phyla found with each database using NovaSeq metagenomics. Pie charts showing (A) the proportions of types of DNA within the Novaseq metagenomics method. (B) Pie chart showing the phyla found using Illumina Shotgun metagenomics in combination with the COI curated marine invertebrate database, (C) the marine Nuclear DNA NCBI-nt database (D) the marine mitochondrial NCBI-nt database and (E) the complete marine NCBI-nt database.

Of the reads that were used for taxonomic classification of the NovaSeq metagenomics dataset, 67.85% (5.775.720 reads) could be assigned to the nuclear fraction of the DNA whereas 7.08% (602.684 reads) was assigned to mitochondrial DNA (Fig. 2A). Between the taxonomic assignment of the nuclear (Fig. 2B) and mitochondrial fraction (Fig. 2C), the nuclear fraction represents more Annelida whereas the mitochondrial fraction represents more Mollusca. In addition, the nuclear fraction represents more phyla that are present with <1%. Within the comparison of alignments with the curated COI marine invertebrate database, the amount of Annelida was 14.32% lower with a total of 5.08% (Fig. 2E). The proportions between phyla obtained with the complete marine NCBI-nt data (Fig. 2D), was used for the diversity comparison between methods, as this covered the most diversity. However, the proportions between phyla obtained through NovaSeq were very dissimilar to the proportions observed in the metabarcoding methods (Fig. S3).

### Alpha diversity obtained with the three DNA-based methods

**Figure 3:**
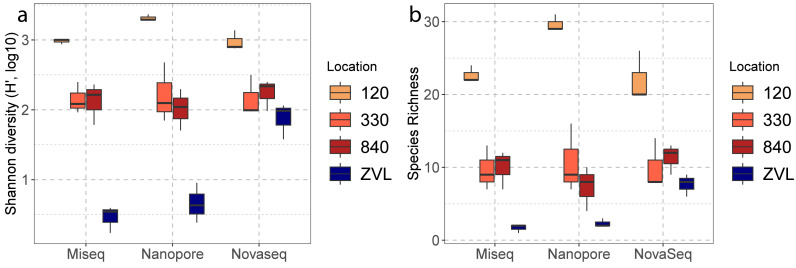
Boxplots of alpha diversities between locations of each DNA based method. The color represents the different location 120, 330, 840 and ZVL. The facets represent each DNA-based method, Amplicon, Illumina MiSeq and Nanopore. Panel (A) represents the number of species (species richness) and panel (B) represents the Shannon diversity for each location and DNA-based method.

A two-way ANOVA showed a significant interaction effect between sequencing techniques (Illumina MiSeq, Nanopore, NovaSeq), and location (120, 330, 840 and ZVL) for species richness (*F* = 4.34, *p* < 0.01). A main effect was also observed for location (*F* = 98.79 p <0.01) and no significant difference was found between methods (*p* = 0.23). *Post-hoc* analysis using a Tukey’s HSD test showed a significant difference between location 120 and all other locations (Table S4), where 120 had the highest richness (Fig. 3A). A significant difference was also observed between ZVL and all other locations (Table S4), where ZVL had the lowest richness. No significant difference was found between location 330 and 840 (*p* = 0.97). As for the interaction effect, the NovaSeq showed no significant different effect between ZVL and location 330 and 840. A two-way ANOVA showed a significant interaction of Shannon indices between sequencing techniques and locations (*F* = 7.67, *p* < 0.01, Table S4). The main effects were only significant for the factor location (*F* = 104.49, *p* < 0.01, Table S4) and for the factor method (*F* = 7.31, *p* ≤ 0.05). *Post-hoc* analysis using a Tukey’s HSD test showed significant differences in Shannon indices between location 120, and all other locations (Table S5), where 120 had the highest Shannon index (Fig. 3B). With the exception of the NovaSeq analysis, a significant difference was also found between ZVL and all other locations (Table S4), where ZVL had the lowest Shannon index (Fig. 3B). Overall, these results highlight that all sequencing techniques similarly observe alpha diversity between locations, except for the richness and Shannon of the Novaseq shotgun metagenomics in location ZVL as more species were observed than the metabarcoding approaches. Shannon indices were also higher for NovaSeq for the low diversity location ZVL.

### Beta diversity obtained from the three DNA-based methods

**Figure 4:**
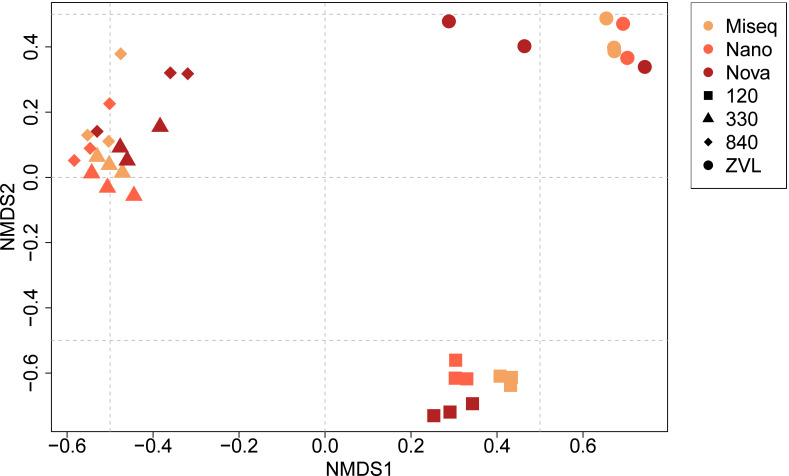
NMDS ordination plot (Bray–Curtis dissimilarity) of similarities of communities between samples and DNA based method. Colors indicate the different DNA based methods whereas the shapes indicate the different locations on species level (log10 transformation).

The PERMANOVA demonstrated significant differences in macrobenthic community compositions between locations (*F* = 16.05, *p* < 0.01) and between techniques used (*F* = 3.25, *p* < 0.01) (Table S5, Fig. 4). In addition, a significant interaction effect was observed between location and the DNA-based approach used (*F* = 2.48, *p* < 0.01), indicating that benthic community composition and location depend on the DNA-based method used and the other way around (Table S5). *Post-hoc* pairwise PERMANOVA tests showed a significant difference in community composition between all locations (*p* < 0.01). Surprisingly, *post-hoc* analysis showed no significant difference in community composition between either sequencing technique. The NMDS plot showed a clear clustering for each location, except for locations 330 and 840 (Fig. 4). The plot also illustrates that location was responsible for the biggest contrasts in community composition, indicating that the location explained most of the variation between community compositions. Similarly, the NMDS plot also indicated that NovaSeq has a different community composition in some locations, particularly within location ZVL. An interaction effect was also observed, indicating that the effect of sequencing platform choice on species composition depends on the community present. Sequencing platforms choice did not affect the species composition for Illumina MiSeq and Oxford Nanopore metabarcoding, as these approaches clustered together within each location.

### Comparison of DNA-based methods for assessing location-specific species composition

**Figure 5:**
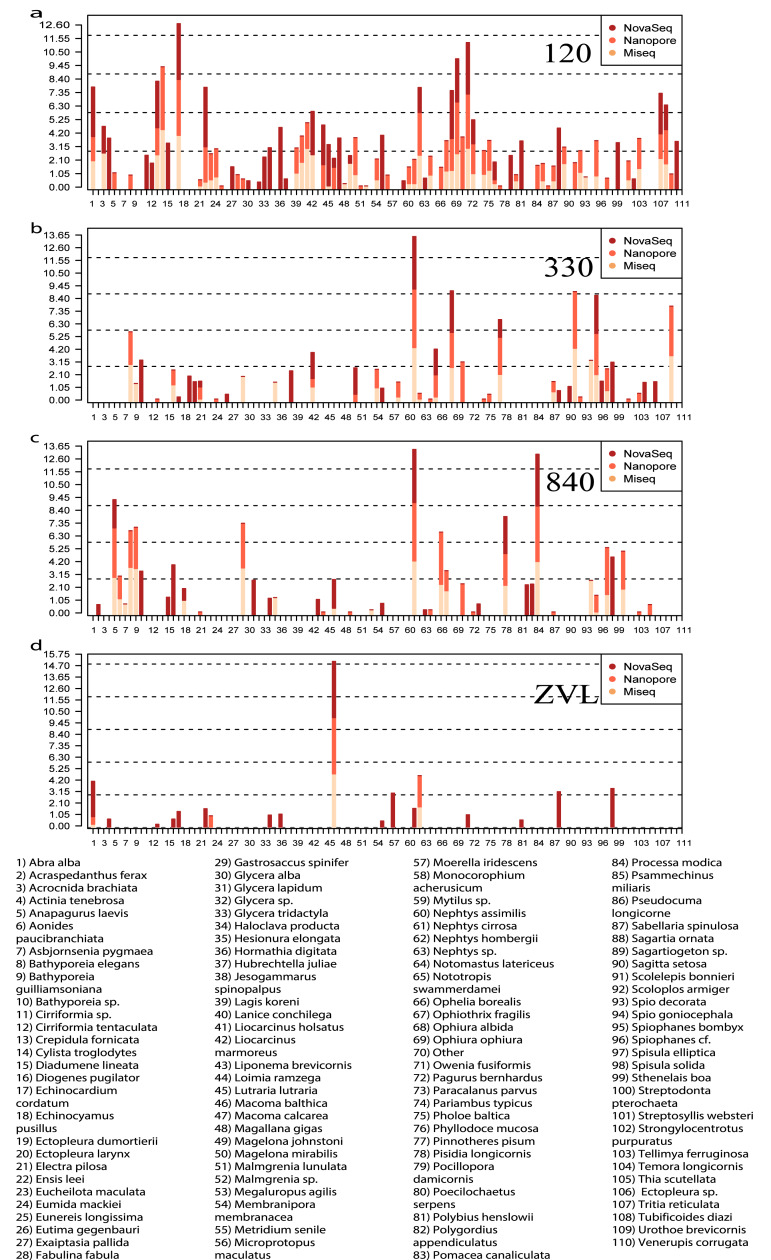
Relative read abundance of each species found between locations and DNA-based method. Bar plot showing the relative read abundance (log_10_) of all species found. Colors represent the different DNA-based methods. (A) The relative read abundance of location 120, (B) the relative read abundance of location 330, (C) the relative read abundance of location 840 and (D) the relative read abundance of location ZVL.

All three DNA-based methods (Fig. 5) shared 25 species and an additional 29 species were shared between just the metabarcoding methods (Fig. 5, Fig. S4). At location 120, 23 species were shared between the two metabarcoding methods whereas only 12 species were shared between all three DNA-based methods (Fig. 5, Fig. S4). The Oxford Nanopore metabarcoding data contained seven unique species for all locations (Fig. 5B, Fig. S4). Interestingly, NovaSeq contained 38 unique species for all locations, and most of these unique species were found in all locations (Fig. 5B, Fig. S4). Several species such as, *Crepidula fornicata*, *Eumida mackiei* and *Magelona mirabilis*, occurred at location 330 using the Oxford Nanopore dataset but with less reads than in location 120 (Fig. 5B). However, these species were only present in location 120 using the MiSeq dataset. Nevertheless, all DNA-based methods detected unique species with each method.

### Comparison of species presence and abundance between DNA-based methods and morphology

**Figure 6:**
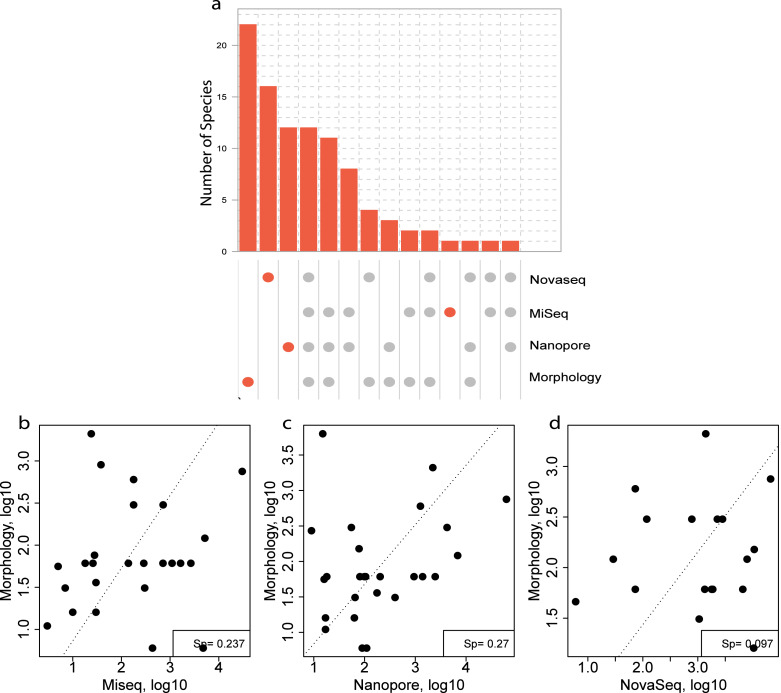
Comparison of species overlap and relative abundance between morphology and DNA-based methods. (A) UpSet plot showing the overlapping and unique species found using each DNA-based method in all the morphologically identified samples. Where the orange dots refer to unique species per method, and the gray dots shared among the respective methods. A Spearman correlation of the overlapping species abundance between morphology and (B) Illumina MiSeq metabarcoding (C) Oxford Nanopore metabarcoding and (D) NovaSeq shotgun metagenomics.

For the morphological identification, one biological replicate was available (120B, 330C, 840C and ZVLA) for each location. This resulted in the identification of 56 species. A total of 39, 13, 10 and three species were identified at locations 120, 330, 840 and ZVL, respectively (Table S1). Of the 56 species identified, 22 species were identified using morphology only (Fig. 6A, Table S7). Of these 22 species, 13 did not have a reference sequence available in the chosen reference database (Table S7, coloured in red). In addition, nine of the species that did not have a reference sequence available belonged to the phylum Annelida (Table S7), which are known to have low primer efficiency and are therefore harder to include in COI reference databases (Carr et al., 2011). Of the 32 species that were identified using both DNA-based methods and morphology, 12 species were identified using all three DNA-based methods (Fig. 6A), 11 species were identified using the metabarcoding-based methods only (Fig. 6A). Lastly, eight species that were identified morphologically were identified only with the NovaSeq metagenomics method whereas an additional three (Nanopore) and two (MiSeq) were unique for the metabarcoding method (Fig. 6A).

Three species were identified with all three DNA-based methods (Fig. 6A), that were not identified with morphology and an additional eight were identified using only the metabarcoding methods. These eight species included *Scolelepois bonnieri* and *Cylista troglodytes* which represented a substantial amount of the identified reads (Table S6). This indicated that the DNA-based methods can identify species that are missed using morphological identification. Oxford Nanopore identified an additional nine species that were unique (Fig. 6A) but found as singletons (Table S6).

A Spearman rank correlation between morphological size class corrected counts and relative read abundance between each DNA-based method across all locations showed the highest positive correlation with both the metabarcoding methods (Sp = 0.237 (MiSeq), Sp = 0.27 (Nanopore) (Figs. 6B–6D). However, the metagenomics method only showed a weak correlation (Sp = 0.097) with the size class corrected morphological counts (Fig. 6D).

## Discussion

We compared three different DNA-based methods: two amplicon-based metabarcoding approaches (MiSeq, Nanopore) and a shotgun metagenomics approach (NovaSeq). We assessed the robustness between techniques in terms of alpha and beta diversity to understand the suitability for macrobenthos monitoring. We have demonstrated that the two metabarcoding methods showed a similar diversity richness and Shannon as well as location-specific species compositions. The NovaSeq metagenomics method also showed similar alpha diversities, but the community composition was slightly different, mostly due to taxonomic assignments that were uniquely found with this method. Interestingly, 22/56 species found in the morphological dataset were not identified with molecular techniques and shows that more than half of the species were identified. Nevertheless, all methods showed that most species were shared within each location.

### Macrobenthos communities are highly similar using metabarcoding despite using different sequencing platforms, platform specific considerations remain

No difference was found between alpha and beta diversity when comparing a standardized lab protocol with Illumina MiSeq sequencing (Van den Bulcke et al., 2023) to Oxford Nanopore metabarcoding. Furthermore, both methods identified similar species composition and community structure at each location and generally, shared similar ASVs/consensus sequences and proportions of phyla. Therefore, this study clearly demonstrated that Oxford Nanopore and Illumina MiSeq metabarcoding are equally suited for monitoring macrobenthos biodiversity. This is in line with recent comparisons (Chang et al., 2023; Van Der Reis et al., 2023; Voorhuijzen-Harink et al., 2019) showing that the Oxford Nanopore datasets are overall comparable with Illumina MiSeq results. This study demonstrates that, even for high-diversity samples, both sequencing platforms perform comparably in terms of macrobenthos diversity assessments, however platform specific considerations remain, including bioinformatics pipelines and sequencing error rates, that may impact species detection and read count estimations. Oxford Nanopore sequencing identified some species that were uniquely present in this dataset. These exclusive taxa were found mostly in very low relative read abundance and did not influence the alpha and beta diversity. These detections could be explained as an effect of the stochastic nature of PCR that is observed in each dataset (Van der Loos & Nijland, 2021). Indeed, previously reported assessments of reproducibility of laboratories showed minor variations between detected species (Van den Bulcke et al., 2021).

Another potential explanation for these differences is that the Oxford Nanopore chemistry and its protocols might be more prone to ‘barcode leakage’ (Framst et al., 2024). The Oxford Nanopore dataset contained some species with only several reads in one of the replicates, while in another location these species were abundant as is the case for example *Crepidula fornicata* and *Eumidia mackei*. Even though a simple correction of barcode leakage was used for this study (as presented in the materials and methods), it is possible that tag jumping, or barcode leakage was still a problem for this dataset. Although this could not be confirmed as no positive control was considered, several independent projects within the same lab that used the same barcoding protocol had similar issues. After routine testing, this problem was found to be related to the temperature at which barcoded samples were pooled for the Oxford Nanopore sequencing library. Barcode leakage problems have been reported often in metabarcoding studies (Beentjes et al., 2019; Van der Loos & Nijland, 2021). Therefore, it is important to consider protocols that minimize the possibility of barcode leakage. This includes using negative and positive controls at each stage, minimizing the handling and amplification of tagged products, or correcting for a crossover of tags between samples (Beentjes et al., 2019). Similar to other platforms, Oxford Nanopore uses two cycles of PCR, one for the amplification of the region of interest and a second PCR for the barcode attachment using Oxford Nanopore-specific protocols and kits for barcoding. The protocol used for this study could be optimized in such a way that individual samples are amplified with already tagged initial barcodes to circumvent the second PCR step (Srivathsan et al., 2021) or by using amplicon-free barcode kits (Toxqui Rodríguez, Vanhollebeke & Derycke, 2023; Van Der Reis et al., 2023).

The bioinformatics pipeline used in this study could also be responsible for the small differences observed between the metabarcoding results. The 10.4.1 flow cells were used, which greatly improved the quality of the metabarcoding data (Zhang et al., 2023). Nevertheless, clustering and consensus building of the data with bioinformatics processing might also result in overlooking some elements of the biodiversity (Brandt et al., 2021). Therefore, the reads that were not included in a cluster for consensus building were also considered when they met the threshold for taxonomy assignment. This inclusion may have led to the detection of additional species within the individual samples, although this is unlikely since the singletons did not contain any additional species (Table S8).

Despite these minor differences between metabarcoding methods, this did not lead to significant differences in commonly used biodiversity indices. Therefore, this study suggests that Oxford Nanopore and Illumina MiSeq metabarcoding are highly similar for macrobenthos monitoring. However, platform specific challenges need to be considered, as Illumina generally provides high-accuracy short read data effective for fine scale taxa identification. In contrast, Nanopore offers sequencing real-time and long-read reads albeit typically with a higher error rate resulting in more intensive error correction during downstream analysis.

### NovaSeq Shotgun sequencing identified the most method specific species

The primer and PCR-free shotgun metagenomics method yielded similar alpha diversities compared to the metabarcoding methods but resulted in a slightly different community composition between methods within location. These different community compositions between methods were mostly due to additional species that were only found using the metagenomics method. Nevertheless, most species that were detected by NovaSeq metagenomics were also detected by the two metabarcoding methods suggesting that either method can describe the general community equally well.

This study, therefore, reflects current environmental metagenomics studies that show equal or higher levels of biodiversity compared to amplicon-based approaches (Bista et al., 2018; Garlapati et al., 2019; Monchamp et al., 2022; Paula et al., 2022). However, these studies, were not targeting a certain taxonomic group and therefore, reflected a wider spectrum of biota. In contrast, this study aimed to test whether metagenomics is suitable for specifically species/level macrobenthos biodiversity monitoring and therefore the data presented are not directly comparable to other metagenomics studies.

The highest number of uniquely found species was found with the metagenomics method, which was especially apparent in the low diversity samples (ZVL). The most straightforward explanation would be contamination between the high and low diversity samples, as these species were not found in the morphological analysis. Although molecular methods are always prone to cross contaminations in the lab (Van der Loos & Nijland, 2021), it is in this case less likely since part of the extractions used in this study for ZVL previously were also used in other studies (Van den Bulcke et al., 2023) and did not experience similar problems.

Alternative explanations for these findings are (1) that misidentification could have occurred due to the use of a database (NCBI) with mislabelled references (Sinniger et al., 2016) or (2) false positive detection of species that are genetically similar but do not occur in the North Sea as is the case for *Actinia tenebrosa* (Ayre, 1985). Furthermore, (3) the species is present but were not picked up with the metabarcoding method because these species did not have a curated COI sequence. As most of the additionally found species with the metagenomics approach belonged to cnidarians, it is possible that during morphological identifications only part of the animal was present in the initial sample and was therefore also never recorded for the morphological analysis (Lobo et al., 2017).

Although the complete NCBI reference database with relevant species was used, still only 0.35% of the metagenomics reads were assigned to a species. Reference databases that contain environmental sequences are mainly focused on genetic regions that are popular for metabarcoding (Weigand et al., 2019) and references of full (mito)genomes are still in their infancy (Blasiak et al., 2020; Leray, Knowlton & Machida, 2022), it is not surprising that such a low percentage of reads are assigned. Thus, the present findings align with previous research that suggest that shotgun sequencing metagenomics is hardly feasible for environmental studies (Ficetola & Taberlet, 2023; Quince et al., 2017). These results also indicate the necessity to improve reference databases, particularly for full (mito)genomes of macrobenthos, as the limited availability of references largely contributed to the low number of reads that are assigned.

Similarly, there was a weak correlation between species that were identified with the metagenomics approach and the morphologically identified species when correcting for size class. Unfortunately, no biomass data was obtained from the morphologically identified samples, which would be a more appropriate representation of the actual abundance. Nevertheless, a better correlation was found with the metabarcoding data, which is in contrast with findings that suggest a better correlation between found metagenomics reads and biomass (Bista et al., 2018). Since in the study by Bista et al., the complete mitogenomes of all species in the mock community was available, the lack of correlations in this study further suggests that the metagenomics method is presently not feasible for reliable DNA-based monitoring of macrobenthos biodiversity. In the future, focusing on reference databases and especially databases that contain complete (mito)genomes will greatly improve detection rates and improve DNA-based monitoring.

### Mismatches between morphology and DNA based methods not only linked to reference sequence availability

As typical for metabarcoding studies, several species were found with metabarcoding that were not reported during the morphological analysis. This is not surprising as these species are either missed during morphological sorting or can only be identified to a higher taxonomic level (Lobo et al., 2017; Robinson et al., 2022). For this study this was apparent for the Anthozoa *Cylista* that was found with high read count (>13,000 reads), whose tissue becomes hard or impossible to recognize when not intact.

Most taxa were detected using both morphology and a DNA-based technique, but there were 22 species that were not identified using any DNA-based method. This was especially apparent in the high diversity samples. Of these, 13 species could not be identified due to incompleteness of the curated database, which especially in relation to marine invertebrates, has often been reported as a limiting factor (Aylagas et al., 2016; Günther et al., 2018; Steyaert et al., 2020; Willassen et al., 2022). Several efforts have been made to improve (Lavrador et al., 2023) and increase the coverage of these databases (Leray et al., 2018; Radulovici et al., 2021). Approximately 22–43% of all European marine species have reference sequences available in the widely used reference database BOLD (Weigand et al., 2019). Multiple studies in the North Sea have emphasized the importance of enhancing these databases (Christodoulou et al., 2021), indicating a need for sustained long-term efforts. To improve the reference database used here, much effort has been put in vouchering as many species as possible by collaborating internationally with molecular scientists (https://northsearegion.eu/geans/about/). However, after morphological sorting of samples of this study, samples were preserved in formaldehyde which is known to hamper DNA extraction from these specimens therefore a one-on-one comparison was not possible. In addition, of the 13 unidentified species due to incompleteness of the reference database, 7 could be found in the COI NCBI database (manual search, 2025), suggesting that the curated database may be less complete in terms of species presence, but was on the other hand referenced with a voucher species and could therefore be confirmed as the correct identification.

However, as nine species did have the potential to be picked up with the used reference database, more factors should explain mismatches between methods. One explanation is the procedure in the field as small differences may occur in species composition between the grabs. 12 out of the 22 species that were not identified with any DNA based method, had an abundance of only one specimen in the morphologically assigned grab sample. Although others with only one morphological assignment were identified (see Table S6), four species that were not found in the comparison, (*Aonide paucibranciata, Nototrips Swammerdamei, Phyllodoce mucosa* and *Streptodonta pterochaeta*) did occur in other replicas of at least one of the DNA based methods. This suggesting that small differences between grabs explain the mismatch to a certain extend.

During lab procedures biases can also occur, for example primer efficiency have also been often reported to influence metabarcoding results (Van der Loos & Nijland, 2021). Of the 13 species that were missing in the reference database, nine species belonged to Annelida. Annelida, and especially certain Polychaeta, are known to be difficult to sequence using the conventional markers because this group has high variation within the COI region and therefore has lower primer binding efficiency (Carr et al., 2011). This have caused the COI reference databases to be biased towards a lower representation of Annelida. Contrariwise, as the primer efficiency is lower for Annelida, it is likely that the chosen COI primer is not representing the taxonomic group well. This is emphasized by the fact that an additional five annelids were not detected with a DNA based method while they were represented in the reference database. Using multiple markers that are more specific to certain taxonomic groups might therefore greatly improve the capability to detect species from metabarcoding methods. However, consequently this may increase laboratory time and costs (Cordier et al., 2019; Gielings et al., 2021; Meyer et al., 2021). Metagenomic methods can also improve the detection of annelids as this technique does not rely on the amplification of specific regions. Nevertheless, the metagenomics method in this study did not result in the retrieval of more annelids.

Other factors such as the taxonomic assignment of the species using the algorithms of RDP or selecting the best scoring match per query can also be of influence as the size and training set of especially RDP influences the sensitivity of detecting certain species (Ritari et al., 2015). Finally, the taxonomic assignment of the species can be wrong. In this case however, this is less likely as this survey is conducted by experts and samples were quality controlled (see Derycke et al., 2021) suggesting that this had little influence on the results.

In conclusion, although DNA-based methods missed 22 species compared to morphological analysis, the DNA-based methods detected additional species that otherwise remained undetected. This is in line with previous reports, also confirming that DNA-based methods and morphology should remain complementary (Cahill et al., 2018; Kelly et al., 2017; Meyer et al., 2021). However, improvements to the databases, the use of multiple replicates and the choice of multi-marker approach for specific taxonomic groups might further improve detection sensitivity and at some point, may surpass morphological identification.

## Conclusions

In recent years, there has been an increased interest in implementing DNA-based tools into routine biodiversity monitoring practices. To achieve this, standardized protocols are necessary to ensure reproducibility and data robustness across studies and regions, particularly as sequencing technology is evolving rapidly (Van den Bulcke et al., 2023).

In this study, we showed that similar alpha and beta diversity patterns were found regardless of the metabarcoding platform used. Thus, Illumina MiSeq and Oxford Nanopore sequencing results are, at this stage, highly similar and can both be used to monitor macrobenthos biodiversity, although platform specific challenges remain. In addition, NovaSeq metagenomics has the potential for environmental monitoring and showed similar communities. However, metagenomics at this stage does not surpass metabarcoding methods, as it did not improve representation of abundance data, nor did it identify more species that are not efficiently amplified by the COI primer. Incomplete reference databases still hamper detection of metagenomics sequences but mismatches between morphological and DNA based findings are also influenced by fieldwork choices, primer biases and bioinformatics choices. These findings demonstrate that portable, real-time Oxford Nanopore sequencing is ready to be integrated for standard monitoring practices and emphasize the importance of improving sequence reference databases to implement and enhance metagenomics methods for robust and harmonized monitoring practices.

##  Supplemental Information

10.7717/peerj.19158/supp-1 Supplemental Information 1 Morphological observations of each species for each location Columns size class indicate the size class of each species. Size class corrected data were calculated by multiply the average size class with the number of individuals.

10.7717/peerj.19158/supp-2 Supplemental Information 2 Volumes of initial material for extraction =Start volume of BULK material for each extraction. Samples labeled DNA were used directly as DNA extracts.

10.7717/peerj.19158/supp-3 Supplemental Information 3 Read counts before and after processing for each DNA based method and NCBI hits

10.7717/peerj.19158/supp-4 Supplemental Information 4 2-way ANOVA between location and DNA based method Yellow is non-significant observations.

10.7717/peerj.19158/supp-5 Supplemental Information 5 PERMANOVA of the NMDS

10.7717/peerj.19158/supp-6 Supplemental Information 6 Presence absence of each species in each DNA based method, to which Phylum they belong and the read count

10.7717/peerj.19158/supp-7 Supplemental Information 7 Species not found in any DNA based method Represents whether or not the species was present in the GEANS macrobenthos database.

10.7717/peerj.19158/supp-8 Supplemental Information 8 Singletons used for species identification of Nanopore method The species that were found in the fraction of reads that remained Singleton after processing the Nanopore reads with Decona.

10.7717/peerj.19158/supp-9 Supplemental Information 9 Rarefaction curves of the metabarcoding methods (Illumina MiSeq, Oxford Nanopore) of each location

10.7717/peerj.19158/supp-10 Supplemental Information 10 ASV level comparison between metabarcoding methods Comparison of the ASVs (MiSeq) and consensus sequences (Nanopore) that match on sequence level (97% identity). Table in (A) represents the percentage of matching Nanopore consensus sequences with MiSEq ASVs, and from those matching sequences the percentage of differently assigned taxonomies. The pie chart represents what these differently assigned taxonomies are for the nanopore dataset. Table in (B) represents the percentage of matching Illumina MiSeq ASVs with Nanopore consensus sequences, and from those matching sequences the percentage of differently assigned taxonomies. The pie chart represents what these differently assigned taxonomies are for the MiSeq dataset

10.7717/peerj.19158/supp-11 Supplemental Information 11 Pie charts of the found phyla for each DNA based method (A) Illumina MiSeq metabarcoding (B) Nanopore MinION metabarcoding (C) Illumina Novaseq shotgun metagenomics (using the complete NCBI-nt (marine) database.

10.7717/peerj.19158/supp-12 Supplemental Information 12 Venn diagram of species presence between DNA-based methods and locations Venn diagram of the overlapping and unique species between DNA based method with all species from all replicates Diagram (A) represent the overlap between methods in all locations, (B) location 120, (C) location 330, (D) location 840 and (E) location ZVL.

## Additional Information and Declarations

### DNA Deposition

The following information was supplied regarding the deposition of DNA sequences: Marine data archive

### Data Availability

The following information was supplied regarding data availability: The raw data, processed data and the used scripts including descriptive metadata are available in the Marine data Archive: – MiSeq: Doorenspleet, K.; Mailli, A. A.; van der Hoorn, B.; Beentjes, K.; De Backer, A.; Derycke, S.; Murk, A.; Reiss, H.; Nijland, R.; Wageningen University and Research; Nord University; Naturalis Biodiversity Center; ILVO Marine Research; (2024): Illumina MiSeq dataset for molecular macrobenthos biodiversity monitoring comparing Oxford Nanopore and Illumina based metabarcoding and metagenomics. Marine Data Archive. https://doi.org/10.14284/682. – Nanopore: Doorenspleet, K.; Mailli, A. A.; van der Hoorn, B.; Beentjes, K.; De Backer, A.; Derycke, S.; Murk, A.; Reiss, H.; Nijland, R.; Wageningen University and Research; Nord University; Naturalis Biodiversity Center; ILVO Marine Research; (2024): Oxford Nanopore dataset for molecular macrobenthos biodiversity monitoring comparing Oxford Nanopore and Illumina based metabarcoding and metagenomics. Marine Data Archive. https://doi.org/10.14284/683. – NovaSeq: Doorenspleet, K.; Mailli, A. A.; van der Hoorn, B.; Beentjes, K.; De Backer, A.; Derycke, S.; Murk, A.; Reiss, H.; Nijland, R.; Wageningen University and Research; Nord University; Naturalis Biodiversity Center; ILVO Marine Research; (2024): Illumina Novaseq dataset for molecular macrobenthos biodiversity monitoring comparing Oxford Nanopore and Illumina based metabarcoding and metagenomics. Marine Data Archive. https://doi.org/10.14284/684. The custom R scripts used for the Data Analysis are available at GitHub and Zenodo: – https://github.com/amalia03/GEANS_Platform_Comparison_Scripts/blob/main/tax_geans.R. – Amalia M. (2025). amalia03/GEANS_Platform_Comparison_Scripts: GEANS_Platform_Comparison_Scripts (v.1.0). Zenodo. https://doi.org/10.5281/zenodo.15000726.
