# Supplementary figures and images for: Advancing molecular macrobenthos biodiversity monitoring: a comparison between Oxford Nanopore and Illumina based metabarcoding and metagenomics
Source: PeerJ. 2025 Apr 14;13:e19158. doi: 10.7717/peerj.19158 (PMC12005195; doi:10.7717/peerj.19158)

## Miseq Rarefaction curves

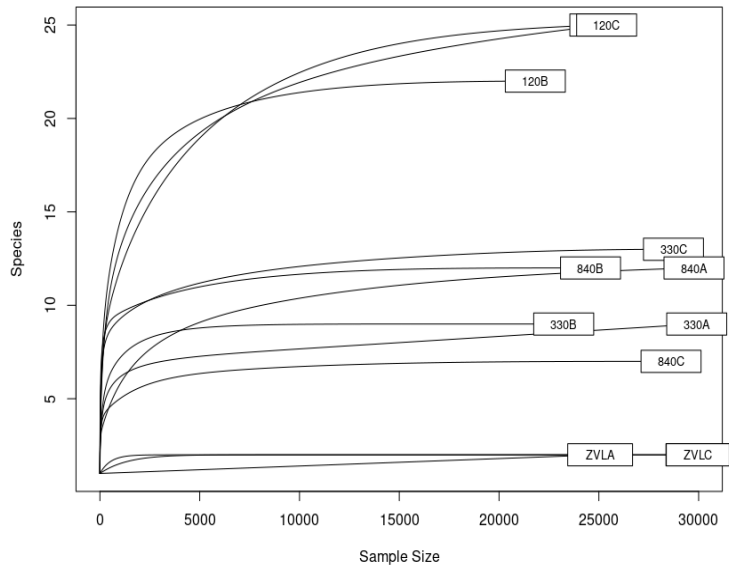

## Nanopore Rarefaction curves

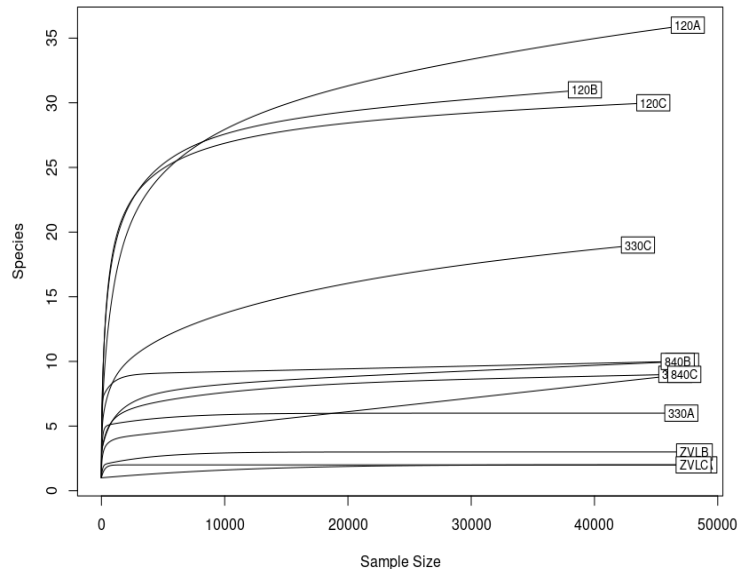

Supplement: Supplemental Information 9 [file peerj-13-19158-s009.pdf]

## a All locations

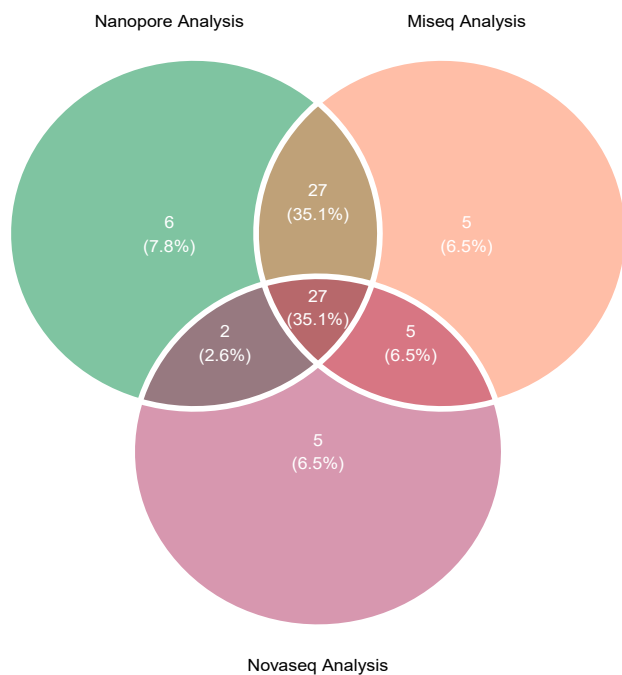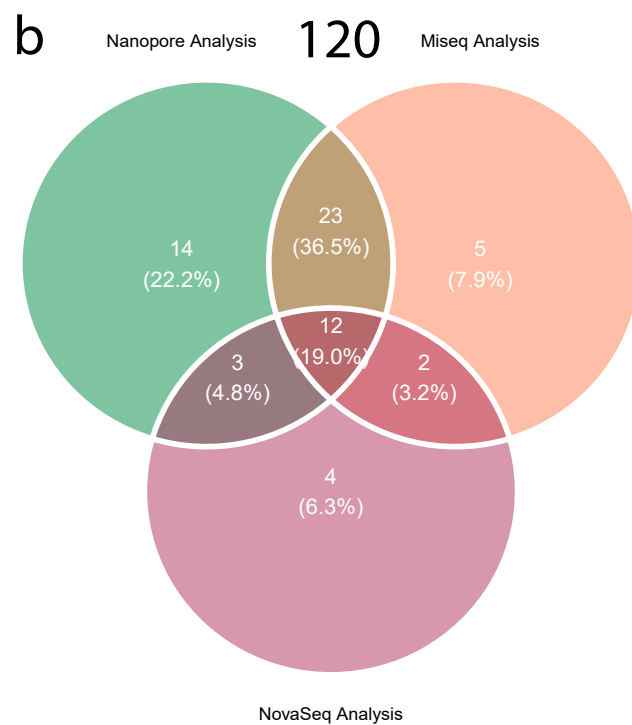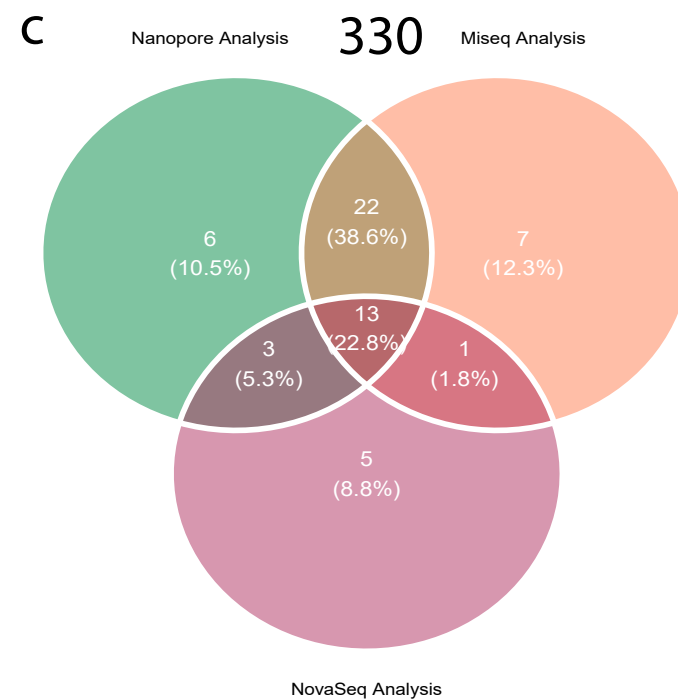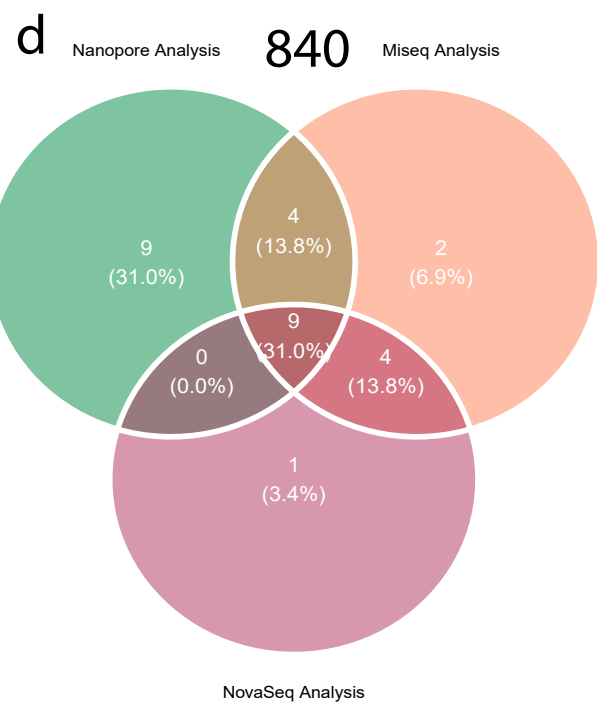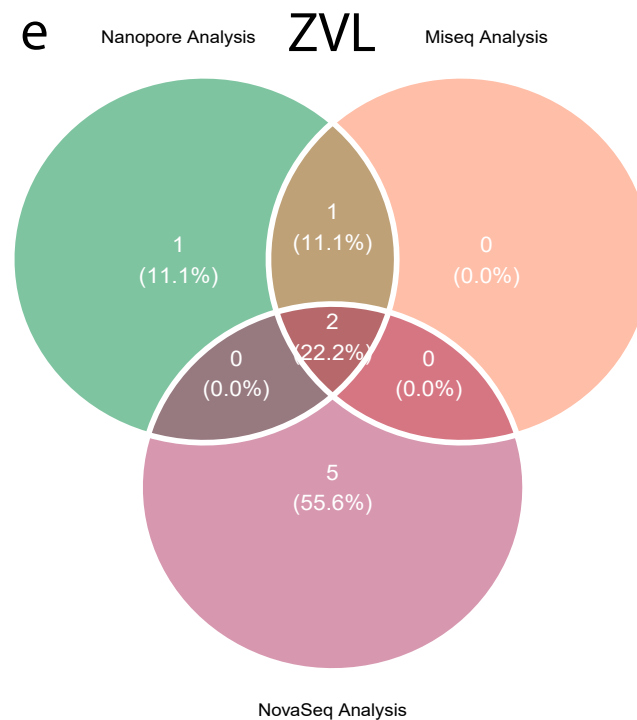

Supplement: Supplemental Information 12 — Venn diagram of the overlapping and unique species between DNA based method with all species from all replicates Diagram (A) represent the overlap between methods in all locations, (B) location 120, (C) location 330, (D) location 840 and (E) location ZVL. [file peerj-13-19158-s012.pdf]
