# Supplementary material for: Advancing molecular macrobenthos biodiversity monitoring: a comparison between Oxford Nanopore and Illumina based metabarcoding and metagenomics
Source: PeerJ. 2025 Apr 14;13:e19158. doi: 10.7717/peerj.19158 (PMC12005195; doi:10.7717/peerj.19158)

a

Total percentage Nanopore consensus sequences matching MiSeq ASVs (>97% identity)	98,86%
From the matching ASVs, the Percentage of differently assigned taxonomies for the nanopore dataset	1.1%

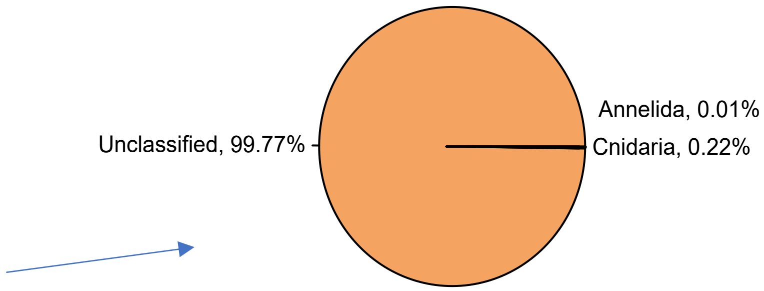

b

Total percentage MiSeq ASV matching Nanopore consensus sequences (>97% identity)	98.79%
From the matching ASVs, the Percentage of differently assigned taxonomy assignments for the MiSeq dataset	1.21%

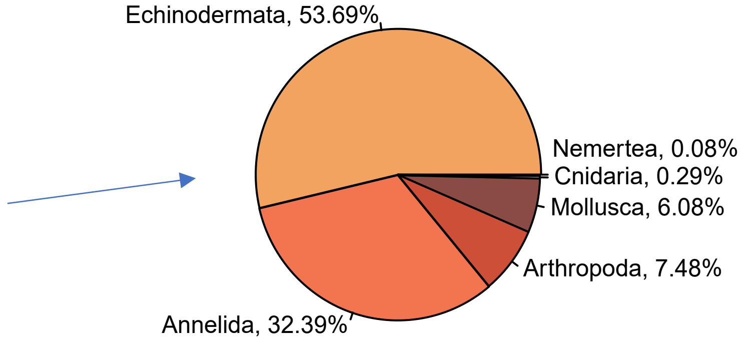

Supplement: Supplemental Information 10 — Comparison of the ASVs (MiSeq) and consensus sequences (Nanopore) that match on sequence level (97% identity). Table in (A) represents the percentage of matching Nanopore consensus sequences with MiSEq ASVs, and from those matching sequences the percentage of differently assigned taxonomies. The pie chart represents what these differently assigned taxonomies are for the nanopore dataset. Table in (B) represents the percentage of matching Illumina MiSeq ASVs with Nanopore consensus sequences, and from those matching sequences the percentage of differently assigned taxonomies. The pie chart represents what these differently assigned taxonomies are for the MiSeq dataset [file peerj-13-19158-s010.pdf]
