# Supplementary material for: Advancing molecular macrobenthos biodiversity monitoring: a comparison between Oxford Nanopore and Illumina based metabarcoding and metagenomics
Source: PeerJ. 2025 Apr 14;13:e19158. doi: 10.7717/peerj.19158 (PMC12005195; doi:10.7717/peerj.19158)
Supplement: Supplemental Information 11 — (A) Illumina MiSeq metabarcoding (B) Nanopore MinION metabarcoding (C) Illumina Novaseq shotgun metagenomics (using the complete NCBI-nt (marine) database. [file peerj-13-19158-s011.pdf]

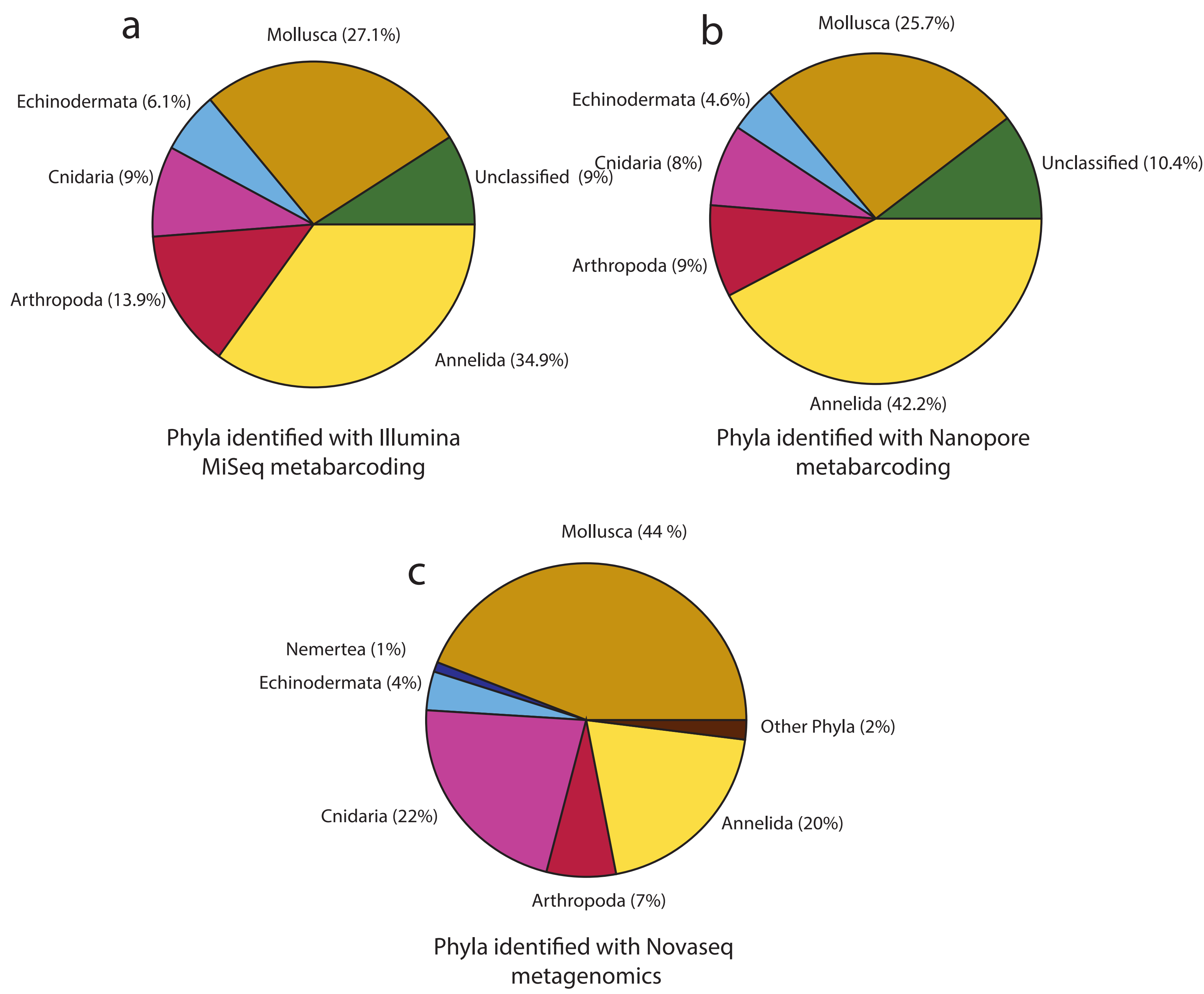
a
Mollusca (27.1%)
Echinodermata (6.1%)
Cnidaria (9%)
Unclassified (9%)
Arthropoda (13.9%)
Annelida (34.9%)
Phyla identified with Illumina MiSeq metabarcoding
b
Mollusca (25.7%)
Echinodermata (4.6%)
Cnidaria (8%)
Unclassified (10.4%)
Arthropoda (9%)
Annelida (42.2%)
Phyla identified with Nanopore metabarcoding
c
Mollusca (44 %)
Nemertea (1%)
Echinodermata (4%)
Other Phyla (2%)
Cnidaria (22%)
Annelida (20%)
Arthropoda (7%)
Phyla identified with Novaseq metagenomics
